# Mechanisms of Microbe–Host Interaction in Crohn’s Disease: Dysbiosis vs. Pathobiont Selection

**DOI:** 10.3389/fimmu.2015.00555

**Published:** 2015-11-19

**Authors:** Ludovica F. Buttó, Monika Schaubeck, Dirk Haller

**Affiliations:** ^1^Chair of Nutrition and Immunology, Technische Universität München, Freising-Weihenstephan, Germany

**Keywords:** IEC, dysbiosis, TNF, Paneth cells, barrier dysfunction, microbiota, Crohn’s disease, IBD

## Abstract

Crohn’s disease (CD) is a systemic chronic inflammatory condition mainly characterized by discontinuous transmural pathology of the gastrointestinal tract and frequent extraintestinal manifestations with intermittent episodes of remission and relapse. Genome-wide association studies identified a number of risk loci that, catalyzed by environmental triggers, result in the loss of tolerance toward commensal bacteria based on dysregulated innate effector functions and antimicrobial defense, leading to exacerbated adaptive immune responses responsible for chronic immune-mediated tissue damage. In this review, we discuss the inter-related role of changes in the intestinal microbiota, epithelial barrier integrity, and immune cell functions on the pathogenesis of CD, describing the current approaches available to investigate the molecular mechanisms underlying the disease. Substantial effort has been dedicated to define disease-associated changes in the intestinal microbiota (dysbiosis) and to link pathobionts to the etiology of inflammatory bowel diseases. A cogent definition of dysbiosis is lacking, as well as an agreement of whether pathobionts or complex shifts in the microbiota trigger inflammation in the host. Among the rarely available animal models, SAMP/Yit and TNF^deltaARE^ mice are the best known displaying a transmural CD-like phenotype. New hypothesis-driven mouse models, e.g., epithelial-specific Caspase8^−/−^, ATG16L1^−/−^, and XBP1^−/−^ mice, validate pathway-focused function of specific CD-associated risk genes highlighting the role of Paneth cells in antimicrobial defense. To study the causal role of bacteria in initiating inflammation in the host, the use of germ-free mouse models is indispensable. Unraveling the interactions of genes, immune cells and microbes constitute a criterion for the development of safe, reliable, and effective treatment options for CD.

## Introduction

Crohn’s disease (CD) is one of the two dominant phenotypes of inflammatory bowel diseases (IBD) characterized by chronic and relapsing inflammation of intestinal segments (1). There is overwhelming evidence corroborating the notion that the pathophysiology of CD is under the control of several contributing factors, including heritable traits, environmental cues, abnormalities in intestinal mucosal barrier integrity (2) and function (3), immune regulation (4, 5), and gut microbiota (6). Exaggerated immune responses are presumably directed against normal commensal enteric bacteria in genetically susceptible hosts. Host genetic susceptibility may be related to defective mucosal barrier function and/or bacterial killing, leading to an overexposure to luminal antigens and to inadequate immunoregulation, resulting in abnormal responses and tissue damage (7–11).

## Genetic Evidence for Microbe–Host Interaction in Crohn’s Disease

Genome-wide association studies (GWAS) have postulated that the heritability of IBD may arise from polymorphisms in associated genes, each contributing in an additive fashion to the overall disease risk (12–15). The most recent GWAS meta-analysis has ascertained 163 susceptibility loci for IBD, revealing similar genetic predispositions in childhood and adult onsets (15). A number of these genetic risk factors are related to innate immunity, most specifically to microbial recognition and its subsequent elimination. The first susceptibility gene described and associated to increased risk of developing CD in Caucasian populations is the nucleotide-binding oligomerization domain 2 (NOD2, also called CARD15) gene. NOD2 gene encodes a cytoplasmic pathogen-recognition receptor (PRR) that recognizes muramyldipeptide (MDP), a component of both Gram-positive and Gram-negative cell walls, and therefore it is thought to play a role in the clearance of intracellular bacteria (16–20).

Additional CD-susceptibility genes that affect bacterial killing include autophagy-related protein 16-1 (ATG16L1) and immunity-related GTPase M (IRGM) (21–26). ATG16L1 gene encodes for an integral protein of the autophagy pathway. Autophagy is an important host defense mechanism for handling intracellular microorganisms and for the response to cellular stress, and it requires the generation of curved membranes to envelop cytoplasmic organelles and intracellular pathogens (27). A working hypothesis is that variants in components of the autophagy machinery may result in reduced pathogen clearance and intracellular homeostasis. IRGM gene encodes for one of the IRG proteins that orchestrate immunity toward intracellular pathogens including autophagy functions (28). While Irgm-deficient mice do not consistently exhibit spontaneous intestinal inflammation, they are much more susceptible to dextrate sodium sulfate (DSS) colitis compared to the WT counterparts, developing acute epithelial injury, loss of Paneth cell granules, altered antimicrobial peptide (AMP) production, and authophagy function of the epithelium (29).

Further CD-susceptibility loci related to aberrant microbial recognition and handling encompass toll-like receptor 4 (TLR4) (30), leucine-rich repeat kinase-2 (LRRK2) (31), and neutrophil cytosolic factor-4 (NCF4) (25). TLR4 is critical for host defense against Gram-negative bacteria since it recognizes lipopolysaccharide (LPS), and it is expressed on intestinal epithelial cells (IECs) and myeloid cells, such as macrophages (MΦs) and dendritic cells (DCs) (32). Two single nucleotide polymorphisms of human TLR4, D299G, and T399I lead to hyporesponsiveness to LPS by interfering with recruitment of TLR adapter molecules, such as myeloid differentiation primary response 88 (MYD88) and TIR domain-containing adaptor-inducing interferon β (TRIF) (33–35). LRRK2 gene is highly expressed on bone marrow-derived myeloid cells, but it is not expressed in IECs (36). Lrrk2-deficient mice do not develop spontaneous inflammation, but they are more susceptible to DSS experimental colitis compared to WT littermates, suggesting a role for innate immune cells in IBD progression (37). NCF4 encodes the p40-phox subunit of nicotinamide adenine dinucleotide phosphate oxidase that is crucial for reactive oxygen species (ROS) production by phagocytic cells in response to microbial infection. Genetic alterations in this CD-susceptibility gene result in abnormal neutrophil and MΦ recruitment, exacerbated cytokine secretion, impaired ROS production, and reduced bacterial clearance in CD patients (38, 39).

A proposed mechanism for the pathogenesis of CD is an exaggerated T (T_H_1/T_H_17) cell response toward luminal microbiota, which results in the breakdown of the mucosal tolerance to enteric bacteria (40). High levels of antibodies against these microbes correlate with disease progression, and T_H_1 and T_H_17 cells are massively recruited to mucosal sites and secrete cytokines, such as TNF-α, IFN-γ, IL-17, and IL-22, which participate to the inflammatory state in CD patients (41–45). GWAS indicated that IL-23 receptor gene and five additional genes involved in T_H_17 differentiation (*IL12B*, *JAK2*, *STAT3*, *CCR6*, and *TNFSF15*) are associated with susceptibility to CD (40). Anti-IL-12/IL-23 antibody therapy, which targets both T_H_1 and T_H_17 cells, is effective in CD (42, 46). Finally, the exact specificity of these T cells, their pathways of activation, and the mechanisms of their dysfunction in CD are still poorly understood. These aspects have been elegantly discussed by others (47, 48) and will not be examined in the current review.

Compelling evidence regarding the marginal role of heritable traits in triggering the onset and causing flares in CD has been provided by GWAS with monozygotic twin cohorts. Monozygotic twins have an (almost) identical genetic make-up, and they also have presumably similar environmental exposure and dietary habits in early childhood. They also have a similar gut microbial composition when healthy (49). Despite familial aggregation being a risk factor for developing IBD (2, 50), germline variation in CD loci of monozygotic twins only accounts for ~25% of estimated heritability, suggesting that the contribution of genetic factors to the etiology of CD is modest (51, 52). Differential exposure to environmental factors (so-called exposome) might account for the low level of concordance in monozygotic twins. Another working hypothesis is that T and B cell receptors (TCRs and BCRs) are not identical in monozygotic twins and that specific receptor rearrangements predispose to an immune-related sensitivity or autoimmune disease (53). In addition, a potential reason for relatively low concordance rates in monozygotic twins may relate to epigenetic changes. The study by Satsangi’s research team provides compelling evidence for epigenic modifications in several regions of the genome of pediatric and adult CD patients (54, 55).

## Sensing of Microbes

Good fences make good neighbors

(Robert Frost, 1914) (56)

The mammalian intestine is inhabited by complex bacterial communities that contribute to the host metabolism and immunity. It remains under investigation how the host maintains homeostasis toward these bacterial populations, discriminating between commensal and pathogenic bacteria-derived signals. A growing body of evidence suggests that IECs contribute to mucosal homeostasis through the integration of microbial signals and interaction with immunocompetent cells (57). Current hypotheses suggest that the breakdown of the intestinal epithelial barrier function leads to increased translocation of food and bacterial antigens, to loss of immune tolerance to commensal bacteria, and to chronic intestinal inflammation, triggering the onset of CD. IECs contribute to tissue homeostasis by maintaining a rigorous spatial separation between microbiota and host, which limits immune activation and promotes induction of tolerance. Specialized IECs may sense the intestinal bacterial community through PRRs, such as TLRs and NOD receptor, and preserve mucosal homeostasis exploiting several strategies to restrict the colonization of resident and pathogenic bacteria and avoid immune overactivation. Processes engaged to this end encompass the secretion of an apical mucus layer by globlet cells (58), the secretion of AMPs by Paneth cells (59, 60), and the transport of secretory immunoglobulin across the epithelial barrier (61, 62). Mucus production has been shown to increase in germ-free (GF) mice exposed to TLR ligands, suggesting that bacteria-derived signals regulate goblet cell functions (58). Paneth cells are specialized cells in the crypts of the small intestine from which they exert a role in crypt homeostasis and maintenance of the intestinal stem-cell niche by secreting AMPs that keeps commensal and pathogenic bacteria at bay and regulate the composition of the gut microflora (63, 64). The expression of AMPs, such as RegIIIγ, is dependent on the recognition of bacterial molecules through Myd88-dependent TLR sensing. Evidence of this mechanism is provided by the proof that epithelial cell-specific Myd88-knockout mice and RegIIIγ-knockout mice loose the segregation between the microbiota and the intestinal surface, leading to a dramatic increase in mucosal-associated bacteria in the terminal ileum (65). Similar loss in spatial organization has been observed in biopsies from CD patients, characterized by lower AMP levels and consequently increased mucolytic bacteria and compromised mucus layer (66–68). Similarly, Muc2-deficient mice develop spontaneous colitis (69).

Small numbers of bacteria that breach epithelial barrier are killed by MΦs or can survive briefly in DCs promoting the induction of IgA by T-dependent and T-independent mechanisms (70). Hence, secretory IgA is selectively induced by DCs loaded with commensal bacteria resulting in local secretion of IgA that limits the penetration and overgrowth of commensal bacteria (71–73). The host may as well produce secretory IgA that is pathogen specific, but it is believed that indigenous bacteria are bound by low-affinity IgA, whereas enteric pathogens are highly coated with IgA (74). Flavell group recently demonstrated that GF mice colonized with highly IgA-coated taxa from IBD patients were much more susceptible to DSS colitis and displayed a bacteria-enriched inner mucus layer compared to GF mice humanized with low IgA-coated bacteria (75). These findings suggest that IgA coating is a defense mechanism that the host engages to mark and distinguish disease-driven bacteria from other members of the intestinal microbiota. The transport of IgA across the intestinal epithelium is modulated by the expression of the polymeric immunoglobulin receptor (pIgR) on the basolateral membrane of IECs. It has been reported that commensal bacteria may activate Myd88- and NF-κB-dependent signaling in IECs and consequently promote the expression of pIgR (62, 76). In addition, microbiota-specific T_H_17 cells may induce epithelial pIgR expression, therefore promoting intestinal IgA secretion and contributing to intestinal homeostasis. This finding is corroborated by the evidence that IL-17 receptor-deficient mice display reduced IgA levels in the gut that can be restored by T_H_17 cell transfer, and increased bacterial translocation and colitis scores upon DSS treatment (77). The serum of CD patients is enriched with high levels of IgA autoantibodies (78, 79). Furthermore, CD patients harbor a stronger humoral immune response against intestinal bacteria than healthy subjects, as shown by the detection of elevated IgG-coated bacteria in CD stool samples (80). This finding corroborates the concept of loss of mucosal tolerance for intestinal bacterial communities in CD patients.

Intestinal epithelial cells are currently emerging as key mediators of inflammatory and immune mechanisms in mucosal tissues. The transcription factor NF-κB has been shown to play a crucial role to control epithelial integrity and immune homeostasis toward gut bacteria in gnotobiotic mice and rats (81–83). Functional proof for the role of NF-kB signaling in the epithelium was provided by an elegant study from Nenci et al. (84). Mice-harboring IEC deletion of *Nemo*, upstream modulator of NF-κB, displayed destruction of intestinal barrier, dramatic translocation of bacteria from the lumen into the mucosa, and developed spontaneous colitis that was Myd88 and TNF receptor-1 dependent (84). Consistent with this protective role of inflammation-related signaling in the epithelium, the absence of TLR-related mechanism conferred increased susceptibility to DSS-induced colitis (85).

Nevertheless, the question remains how much TLR-related microbial sensing confers protection rather than tissue inflammation. It has been proposed that low expression of TLRs at the epithelial level, especially TLR2 and TLR4, is associated with mucosal homeostasis by maintaining a hyporesponsive state toward the presence of commensal bacteria (86–88). Notably, epithelial tolerance to microbial ligands seems to occur immediately after birth in order to promote a stable intestinal host–microbe homeostasis (89). Nevertheless, the high turnover rate of the epithelium may require additional immunosuppressive mediators of the mucosa such as transforming growth factor (TGF)-β and prostaglandin (PG) J2 in order to maintain epithelial cell homeostasis in response to the constant bacterial challenge (82, 83, 90). In the context of infectious or chronic inflammatory conditions, IFN-γ and TNF-α upregulate TLR expression in IECs (91). Consistently, TLR4 is significantly increased on the apical side of IECs throughout the lower gastrointestinal tract of CD patients (86). With respect to the maintenance of mucosal immune homeostasis, it has also been postulated that the expression of individual TLR is limited to specific cell lineages (92), and it is spatially restricted on the apical or basolateral intestinal surface (93, 94). For instance, TLR1, TLR2, and TLR4 seem to be coexpressed on a subpopulation of human and mouse cells located in the intestinal crypt and belonging to the enteroendocrine lineage (92). Analysis of polarized human IECs *in vitro* indicated that TLR5 is expressed on the basolateral surface and from this location triggers the production of cytokines and chemokines in response to flagellin (93).

Macrophages and DCs sense microbial components of the intestinal lumen, providing a key link between the microbiota and epithelial barrier functions (95, 96). The primary role of MΦs is to phagocytose cellular debris and microbes and stimulate lymphocytes and other immune cells to respond to the antigen through the secretion of cytokines and chemokines. Depending on the requirements of the surrounding tissue environment and of the stimuli encountered, MΦs express different biological functions exhibiting a remarkable plasticity (97). Gastrointestinal mucosal MΦs play a crucial role in maintaining gut homeostasis (98). Considering the huge bacterial load present in the intestinal lumen, it is likely that commensals (and pathogens) breach the epithelium. Mucosal MΦs are strategically located adjacent or in proximity of the epithelia and along the lamina propria. Intestinal MΦs are characterized by low production of proinflammatory cytokines but with an intact phagocytic ability (99). Indeed, this MΦ population helps to maintain a low level of inflammation in the lamina propria (state of physiologic inflammation), in order to prevent inappropriate immune responses to microbes. This is possible because intestinal MΦs express low level of TLR2 and TLR4, the two main receptors involved in sensing bacterial cell wall components (100). However, even though they may express TLR1, TLR3, and TLR5–9 to different extents (101), mucosal MΦs do not release proinflammatory cytokines in response to TLR ligands. They express low levels of TRIF, Myd88, and TRAF6 proteins, leading to an inability to phosphorylate NF-κB p65 and MAPKs (98). Intestinal MΦs retain a highly phagocytic activity, but they do not present antigen in normal intestinal mucosa as they lack constitutive expression of the costimulatory molecules CD40, CD80, and CD86, displaying a tolerance-inducing phenotype (98). In contrast, intestinal MΦs from CD patients express high levels of costimulatory molecules (102–104), increased activation of NF-κB signaling and oxidative burst activity (105, 106), high expression of TLR2 and TLR4 (107–110), and secretion of cytokines, including TNF-α and IL-23 (111).

Dendritic cells located in the lamina propria can penetrate the epithelium without disrupting the barrier function and acquire antigens from food or directly sample gut-associated bacteria and take them to mesenteric lymph nodes, where they are presented to CD4^+^ T cells. In contrast to DCs loaded with harmful bacteria that reach systemic secondary lymphoid structures (spleen and lymph nodes) and activate systemic immunity, DCs loaded with commensal bacteria migrate to the MLNs but remain confined in the mucosal lymphoid tissue (112). IECs release mediators (e.g., TGF-β, thymic stromal lymphopoietin, and PG E2) that maintain DCs in a quiescent state and promote the induction of regulatory T cells (113). This mechanism allows the host to develop systemic tolerance in response to commensal colonization which appears compromised in CD patients (114). During inflammation, IL-12 and IL-23 produced by DCs restrain regulatory T cells and promote T_H_1 or T_H_17 effector cells, respectively (42). The crucial role of IL-23 in promoting chronic mucosal inflammation has been emphasized by evidence suggesting that this cytokine, produced by innate immune cells, may inhibit FoxP_3_ Treg cell function and consequently positively modulate T_H_17 differentiation (115).

## Ileitis Phenotype in Mouse Models with Deficiency in Crohn’s Disease-Associated Genes

Genetic polymorphisms associated to CD may alter immune responses to commensal bacteria and mucosal barrier function impacting the microbiota composition in the gut. The possible mechanism for genetic regulation of enteric microbiota include altered Paneth cell function but also altered barrier functions and mucus production, defective secretion of IgA, and altered innate and adaptive immune responses.

Paneth cells are specialized IECs located adjacent to the stem cell zone in the base of the crypts in the small intestine. They are critically involved in host defense against enteric pathogens by secreting AMPs and TNF-α (116, 117). Mutations in genes associated with CD, usually highly expressed in Paneth cells, predispose to the development of ileal lesions in humans (118). In line with this, mouse model with deficiency in several CD-associated genes, including *Nod2*, *Atg16l1*, and *Xbp1*, displays Paneth cell defects and susceptibility to intestinal inflammation (119, 120).

Nucleotide-binding oligomerization domain 2 is a well-acknowledged susceptibility gene for CD (16, 18, 121). NOD2 is thought to play a relevant role in maintaining microbial tolerance at the intestinal barrier (16) and to activate innate and adaptive immunity (63). Three common mutations in the C-terminal leucine-rich repeat region of the NOD2 gene, namely G908R, R702W, and the frameshift deletion mutation L1007, and several other rare polymorphisms have been discovered and associated to increased risk of developing CD. How the NOD2 variants increase susceptibility to CD remains debated. For instance, the frameshift variant L1007 has been shown to decrease NF-κB activity in HEK293 cells stimulated with a number of common bacteria (17). This observation contradicts the evidence that CD clinical specimens are characterized by elevated NF-κB activity (122). One possible explanation is that the mutation in NOD2 leads to a defect in the innate immune response allowing intracellular bacteria to escape the first line of host defense, resulting in an enhanced adaptive response. In support of this hypothesis, elevated proinflammatory cytokine production was detected in splenocytes and blood mononuclear cells from Nod2-deficient mice challenged with TLR2 ligands (123, 124). Furthermore, Nod2^−/−^ mice become more susceptible to colitis as a result of enhanced TLR2 responses characterized by increased production of IL-23 and IL-12 (124). However, the role of NOD2 as negative regulator of TLR2 is controversial since the same L1007 frameshift mutation has been shown to protect mice from systemic infection (and inflammation) by *Enterococcus faecalis* that is a Gram-positive bacterium and therefore sensed mainly by TLR2 (125). Notably, Nod2^−/−^ mice do not spontaneously develop an inflammatory phenotype (63) but exhibit a higher load of commensal bacteria (i.e., *Bacteroidaceae*) in the terminal ileum and Peyer’s patches and a reduced ability to prevent pathogenic bacteria colonization (126–128), suggesting a genotype-driven selection of a pathobiont-enriched microbiota. Similarly, the NOD2 variant L1007 is associated with higher colonization of the intestinal mucosa by the *Bacteroidaceae* in humans (129). It is still not clear whether the observed dysbiosis is the cause or a consequence of the disease, but it is tempting to speculate that NOD2 variants are associated with changes in the composition and load of the commensal microbiota in the terminal ileum that may facilitate disease progression and pathology. In line with this speculation, WT mice develop colitis when recolonized with dysbiotic fecal microbiota from Nod2-deficient mice (130). In addition, crypts and Paneth cells from Nod2^−/−^ mice have attenuated antibacterial activity and decreased expression of α-defensin and cryptidin leading to increased susceptibility to *Listeria monocytogenes* infection *in vivo* (63, 126). These findings are in agreement with the deficiency in α-defensin production observed in CD patients (3, 131, 132).

In the gut, bacterial growth and division contribute to the remodeling of components of the bacterial cell wall, i.e., pepdidoglycan (PGN), by bacterial autolysins, e.g., muramidases and amidases (133). During this process, soluble PGN fragments can be transferred to the bloodstream and initiate innate immune responses through PRRs, including NOD2 and TLR2 (134, 135). Specific classes of bacteria, such as Actinomyces and Mycobactera, use hydroxylases to covalently modify MDP-generating moieties that activate more potently the NOD2 pathway. This evidence raises the intriguing possibility that bacterial components may account for the differential activation of immune cells resulting in detrimental effects on a host immunity already compromised, such as in CD.

Nucleotide-binding oligomerization domain 2 has been reported to account for stem cell protection and to contribute to stem cells regeneration via responses triggered by MDP recognition and leading to the recruitment of NF-κB at the membrane surface of IECs (136–139). Conversely, NOD2 mutant 3020insC, which is associated with CD, shows an impaired ability to activate NF-κB following MDP stimulation *in vitro* (138, 140, 141). In the absence of bacterial invasion into the host cytosolic compartment, it has been postulated that MDP can cross plasma membrane and localize into the cytosol via the plasma membrane transporter, PepT1 (142, 143). During chronic inflammation, such as CD, PepT1 expression has been shown to be upregulated in the colon (144, 145). Nevertheless, a recent study argues against the indication of a role for PepT1 in the development of intestinal inflammation, showing that in animal models resembling Crohn-like ileitis (TNF^deltaARE^) and colitis (IL-10^−/−^, IL-10XTLR2^−/−^, and Rag2^−/−^) and in human intestinal tissues from IBD patients, severity of inflammation correlated with lowered PepT1 expression levels (146).

Nucleotide-binding oligomerization domain 2 is involved in the cellular protection mechanism called autophagy, by directly interacting with the ATG16L1. Since both of these genes are CD-susceptibility loci, it has been proposed that loss of autophaghy-related function is implicated in the pathogenesis of CD. DCs (147), MΦs (148), and epithelial cells (149) containing ATG16L1 and NOD2 variants show defects in antibacterial autophagy. In DCs, these defects are associated with an impaired ability to process pathogens (such as *Salmonella typhimurium*) and present exogenous antigens (such as MDP) to CD4^+^ T cells (147). Murine Atg16l1- and Nod2-knockout MΦs secrete aberrant IL-1β levels in response to LPS (150) and MDP (122), respectively. These findings highlight that CD-susceptibility genes require interaction with environmental (microbial) cues to manifest a disease phenotype. This is further supported by the observation that GF hypomorphic Atg16l1 mice are disease free, whereas colonized mice display aberrant Paneth cells morphology and antimicrobial protein expression, and they are highly susceptible to DSS-induced colitis (119, 150, 151). This phenotype has been recently associated to dysfunctional IECs due to mutations in the autophagy machinery rather than arising from defects in granule formation in Paneth cells (152). In fact, the author exploited IECs-specific Atg16l1-knockout mice to demonstrate that the deletion of the autophagy-related gene is responsible for reduced Paneth cell number, abnormal granule morphology, reduced expression of AMPs, and increased inflammation and systemic translocation of *S. typhimurium* compared with control mice (152). Similarly, CD patients carrying the ATG16L1 T300A mutation show an autophagy-associated defect in Paneth cells with granule abnormalities (153). Exploiting mice with epithelial cell-specific Myd88 deletion, Hooper group demonstrated that only invasive bacteria, such as *S. typhimurium* and *E. faecalis*, can activate autophagy in IECs in a Myd88-dependent fashion (154). In addition, the authors showed that autophagosome formation *in vivo* is TRIF independent, while in RAW MΦs autophagy was reported to be depended on this adaptor molecule (155). Nod2^−/−^ mice do not harbor defects in autophagosome formation, rather lack of the intracellular PRR is associated to an increased number of bacteria within ileal IECs compared to WT mice, as shown by FISH analysis (154). This finding suggests that bacterial breach of the intestinal epithelium triggers autophagy in IECs in Nod2-independent manner. Mice with an IEC-specific deletion of the essential autophagy gene *Atg5* display decreased numbers of autophagosomes but no histological evidence of pathology and an increased number of intracellular *S. typhimurium* (154). These findings highlight the crucial role of IECs as a selective barrier that contributes to maintaining mucosal homeostasis by finely tuned communications with gut microbiota and the luminal environment, peripheral tissues, and the immune system (156).

Multiple cellular stress responses were observed in IBD, including endoplasmic reticulum (ER) and mitochondrial (mit) unfolded protein responses (UPRs). The accumulation of misfolded proteins in the ER and mit lumen of IECs activates several processes, including inflammation and UPR signaling pathways, and the integrated stress response (157, 158). TLR signaling and autophagy cooperate in bacterial sensing actively interacting with cellular stress responses, i.e., UPR. UPR signaling is mainly driven by inositol requiring, ER-to-nucleus signaling protein 1α–X-box-binding protein-1 (XBP1) pathway. XBP1 gene deletion may lead to increased ER stress as a consequence of a defective UPR in secretory IECs (i.e., goblet and Paneth cells), thereby affecting their function (120). While Xbp1-deficient mice are protected from ileitis in GF housing, colonized Xbp1^−/−^ mice develop spontaneous transmural inflammation, resembling Crohn’s ileitis phenotype in humans (120, 159). In particular, mice with Xbp1-deficient IECs develop spontaneous enteritis and display ER stress with high level of the chaperone grp78 and C/EBP homologous protein (Chop), Paneth cell loss, and reduced globlet cell number and size, compromised response to pathogenic bacteria (i.e., *L. monocytogenes*), and they are more susceptible to DSS colitis (120). Overexpression of CHOP in IECs aggravates DSS-induced colitis and impairs mucosal wound healing (160). *In vitro*, XBP1 deficiency induced ER stress that led to a heightened JNK-dependent proinflammatory response of epithelial cells to flagellin and TNF-α (120). The emerging role of ER stress in the progression of CD is supported by the detection of high levels of ER stress markers (i.e., grp78) in ileal and colonic epithelia of CD patients (120, 161, 162). Generation of multiple cellular stress responses, and consequently inflammation, in the intestinal epithelium is multifactorial and includes genetic and environmental factors (163, 164). Paneth cells from patients with quiescent CD and healthy controls carrying the ATG16L1 T300A risk allele are characterized by increased ER stress (153). This finding indicates an interaction between ER stress mechanisms and autophagic pathways. For instance, CD patients may harbor a defect in barrier function which results in overactivation of Paneth cells by bacterial ligands, requiring a higher demand of secretory AMPs, potentially leading to ER stress (165). It is possible to speculate that progression of ER stress in CD patients may be facilitated by the production of ROS induced by the inflammatory cytokine TNF-α (166).

The evidence of barrier dysfunction in CD patients, associated with the inability to achieve mucosal healing or to seal off the damaged epithelium, confers a role of relevance to tight junction proteins as risk factors for disease development (167). Notably, aberrant (high) tight junction-dependent paracellular permeability (168, 169) and exacerbated TNF-α level has been observed in CD patients as well as in their healthy first-degree relatives (170–173). A proposed mechanism is that inflammatory cytokines, i.e., TNF-α, may activate myosin light chain kinase (MLCK) which induces tight junction disruption ultimately leading to cytoskeletal-related barrier defect (174–177). This phenomenon leads to the activation of systemic T cell-mediated immune responses and impaired barrier function (178). A major validation of this molecular mechanism comes from the observation that wild-type mice treated with MLCK inhibitor and mlck-knockout mice are protected from barrier dysfunction (174, 178). In contrast, transgenic mice expressing mlck constitutively (CA-MLCK Tg) displayed increased paracellular permeability within the small intestine and colon, without developing spontaneous disease (179). Intriguingly, transfer of colitogenic T cells into CA-MLCK Tg mice accelerated the onset and severity of colitis (179). This finding suggests that increased gut permeability is insufficient to trigger disease in the absence of other predisposing factors, which most likely include immune dysregulation and altered luminal microbiota.

## What is the Contribution of Microbiota to CD?

The microbial composition in the intestinal tract is considered another potential risk factor in individuals with CD (51, 180). There are several lines of evidence for microbial involvement in IBD. For instance, (i) inflammation occurs in regions with higher bacterial density, such as distal ileum and colon, (ii) GF animals do not develop ileitis (181) or colitis (182), (iii) antibiotics have shown some therapeutic efficacy in IBD patients (183), (iv) the severity of the disease correlates with the bacterial density in the intestinal mucosa, and (v) a large number of studies reported an altered bacterial composition in IBD patients compared to healthy individuals, so-called dysbiosis (184).

## The Gastro Intestinal Ecosystem – Who is There?

The mammalian gastrointestinal tract is habitat to taxonomically diverse microorganisms in very close proximity to the host. The totality of these microorganisms, so-called “microbiota,” includes bacteria, viruses, archaea, and eukaryotes (yeasts, protozoa), and their genes represent the “intestinal microbiome.” The human and murine intestines are dominated by the bacterial phyla *Bacteroidetes* and *Firmicutes* with a minor proportion represented by *Proteobacteria*, *Actinobacteria*, *Verrucomicrobia*, *Tenericutes*, and *Fusobacteria* (185, 186). At lower taxonomic levels, the diversity is very high, with ~100–200 bacterial species per individual (187). Notably, great interindividual differences were observed in the overall microbial community, highlighting the limitation in defining the absolute composition of a “healthy” microbiota (188–191). In the last decades, compelling evidence emerged pointing out the pivotal role of the intestinal ecosystem in defining the host immune homeostasis (e.g., by inducing pro- or anti-inflammatory responses) (192). Therefore, the study of shifts in the intestinal ecosystem is of fundamental importance to understand associations and causalities between gut microbes and immunity.

## Characteristics of Dysbiotic Ecosystems

Under physiological conditions, the microbiota shows both plasticity and high resilience, i.e., upon short-term perturbations (e.g., change in dietary pattern), the microbial composition adapts to alterations in the intestinal milieu, though soon resembles a predisturbance state (189). The microbial ecosystem can also be changed without pathologic consequences for the host and stabilize within a new “alternative state” (193). Therefore, the microbiota is capable of adapting to exposomal factors including diet, smoking, antibiotics, or intrinsic factors like host genetics. Nevertheless, diet seems capable to overwrite the influence of genetic imprint (194–196). Rapid resilience to perturbations is a key requirement for the intestinal homeostasis and the host’s health in order to maintain a health-associated composition of the ecosystem (eubiosis). However, this resilience is lost in some pathologic conditions, like IBD. In coexistence with genetic susceptibility to inflammatory processes – like in IBD patients – the microbiota is causative for disease development and is therefore considered as “dysbiotic” (197).

For different pathologies, there is a strong evidence for the role of “dysbiosis,” defined as an alteration in the ecosystem associated to pathology (180, 198–200). While for CD and UC, the connection between dysbiosis and pathology seems well established; also other pathologies like obesity, diabetes, cardiovascular disease, and even depression or multiple sclerosis have been recently connected to intestinal dysbiosis (198, 201–204)

However, dysbiosis displays several features, which will be in the present review categorized in (i) reduced bacterial diversity, (ii) expansion of pathobionts, (iii) changes in the microbial composition, i.e., increase or reduction in indicator species, and (iv) change in microbial functional capacity (205). Thereby, the appearance of these characteristics may occur solitarily, successively, or simultaneously.

The most widely discussed attribute of dysbiosis is *reduced bacterial diversity*. Based on the numbers of bacterial species and their abundance found within one sample, the alpha diversity can be calculated. Many studies associated lowered bacterial diversity to disease, with the rationale of loss in metabolic redundancy (180, 206–209). Several reports have addressed dysbiosis and decreased diversity in IBD patients’ display compared to healthy individuals (51, 180, 200, 206, 210–214). Furthermore, the ability to outcompete pathogens by a low-diverse microbiota is diminished. In patients who underwent frequent antibiotic treatment, the deteriorated intestinal diversity was shown to increase the risk of infection by opportunistic pathogens, such as *Clostridium difficile* (215). In animal models, pathogens including *Salmonella*, *Citrobacter rodentium*, or enterohemorrhagic *Escherichia coli* fail to colonize in the presence of a diverse, undisturbed microbiot but elicit pathogenic traits if competing strains are missing (216–219). The mechanisms of competitive exclusion may correspond to rivalry for nutrients or virulence modulation of intruding strains (220).

Dysbiosis may also be simplified by linking it to *expansion of pathobionts*, i.e., single strains of the commensal microbiota that outgrow and cause detrimental effects in the host. The term pathobiont was defined by Chow and Mazmanian as “…symbiont that is able to promote pathology only when specific genetic or environmental conditions are altered in the host” (221). While pathobionts are found only in low abundance in a healthy microbial setting, they overgrow in dysbiosis and cause disease in the susceptible (e.g., immune compromised) host. Hereby, specificity in the combination of microbe and host susceptibility is required. In animal studies, it was shown that *Bacteroides vulgatus* induced colitis in HLA/B27-ß2m rats, but not in IL-10^−/−^ mice and even prevented colitis in IL-2^−/−^ mice (222, 223). Early studies regarded *Mycobacterium avium* subspecies *paratuberculosis* as the responsible pathobiont or even pathogen in IBD. However, this hypothesis could not be verified, as summarized by Packey and Sartor (224). In stool samples and mucosal specimens from IBD patients, an increased abundance of *Enterobacteriaceae* is repeatedly observed, and among these the *E. coli* strain LF82 is discussed to be a pathobiont (225–227). The group around Darfeuille-Michaud was the first to describe adherent-invasive *E. coli* (AIEC), which selectively colonize the ileum of CD patients, suggesting that dysbiosis in IBD may also relate to strain-specific virulence factors (6, 228–231). The authors showed that the AIEC strain LF82 is able to persist within MΦs and epithelial cells and selectively colonizes the ileum of CD patients.

Apart from single pathobiont alterations, dysbiosis is in most cases regarded as *shifts in the overall microbial composition*, i.e., simultaneous increased or decreased abundance of certain commensals. Due to the new sequencing techniques and improved databases, great progress has been made in characterizing the intestinal microbiome also in larger cohorts. However, most samples were taken from patients who already underwent some sort of treatment, which exerts changes in the ecosystem and thereby impede conclusive interpretation of findings (232–234). A study from Gevers et al. elegantly solved this issue by picturing the treatment naive microbiome in children recently diagnosed for CD, before shifting the intestinal ecology by different forms of pharmaceutical or nutritional intervention. They described an increase in *Enterobacteriaceae* preceding the onset of CD, an observation made by other studies as well (199, 234, 235). It is worth noting, however, that small inflammatory lesions may result in changes in microbiota composition prior to the diagnosis of disease. Thus, prospective cohorts would be of high value but demanding considering the low incidence of IBD. Among the multitude of studies performed to detect IBD-associated bacterial taxa, little congruence is found between different cohorts. By combining the sequencing data from several studies, Walters et al. showed shifts in the bacterial composition at different taxonomic levels, which were at least partly consistent for several studies and cohorts (227). They showed higher abundance of *Actinobacteria* and *Bacteroides* sp. and a loss of *Prevotella* sp. in CD patients compared to healthy controls. They also described at lower taxonomic level the loss of some *indicator species* in IBD, like *Faecalibacterium prausnitzii*. This health-associated species was found in significantly lower levels in the inflamed intestine compared to healthy specimens and exerts positive immune-regulatory effects on the host (236, 237). Therefore, loss of *F. prausnitzii* is speculated to be an indication for increased IBD risk (211, 236, 237). However, use of just a single indicator species as diagnostic tool may not be sufficient, as IBD-associated strains may be cohort or individual specific. Nevertheless, the novel approach of using the analysis of overall drifts in the intestinal, preferably stool microbiota as a diagnostic tool is a promising new tactic in IBD diagnosis. Walters et al. highlighted some universally valid disease-related shifts, which may be powerful enough to securely diagnose IBD, by combining the sequencing results from several studies (227). By using stool microbiota, this non-invasive approach is even more advantageous.

By great advances in 16S rRNA profiling methods, shifts in the prevailing bacterial members of the intestinal composition can be more easily assessed and discussed. However, most descriptions of intestinal dysbiotic communities fail to take fungal and viral contributions into consideration. Recently Chehoud et al. could show reduced fungal diversity in pediatric CD accompanied by increased *Candida* taxa (238). Norman et al. showed marked differences in the intestinal virome in CD and UC patients compared to healthy controls (239). The main difference was an increase in *Caudovirales* bacteriophages which was not secondary to bacterial dysbiosis, but it is more likely to assume that viral dysbiosis contributes to pathology and changes in the bacterial ecosystem due to a “predator–prey” relationship (240–242). However, studies of the role of other microbial taxa in IBD pathogenesis are awaited and will be essential to unravel dysbiotic patterns.

In addition to bacterial changes in composition and diversity, changes in the *functional metabolic capacities* are characterizing a dysbiotic ecosystem on a functional level. As shown by the human microbiome project, the healthy intestinal ecosystem may be highly different in composition, while its metabolic activity is highly similar (190). An interesting study in IBD patients by Morgan et al. showed a small perturbation of the intestinal composition, though a quite distinct change in microbial function (235). In dysbiotic conditions, microbial pathways for oxidative stress tolerance, immune evasion, metabolite uptake, and carbohydrate as well as amino acid biosynthesis were upregulated. An increase in carbohydrate metabolism and especially changes in the metabolic capacity to utilize fucose were reported in dysbiotic settings in CD patients or animal models of inflammation by others as well (181, 243–245). Metaproteome analysis in a cohort of CD patients also proved a distinct protein signature associated to CD (246). By correlating these functional shifts to the bacterial ecosystem, *Bacteroides*-derived proteins related to survival in challenging environments (e.g., DnaKs and other chaperones) were found overrepresented. Dysbiosis may also be described by changes in the intestinal microbial function and consecutively produced metabolites (247, 248). This leads to the assumption that dysbiosis may be more precisely characterized by changes in the microbial function rather than composition.

Dietary fibers are often associated to be reducing the IBD risk, as fibers are metabolized to short-chain fatty acids (SCFA) by microbes in the distal gastrointestinal tract (249). Those SCFA were found to hamper the growth of pathogens, increase the intestinal barrier function, and serve as energy source for colonocytes (250–255). Furthermore, they also facilitate the generation and differentiation of regulatory T cells in the gut and thereby are important to maintain a homeostatic environment (252, 254, 256). Machiels et al. have reported that UC is associated with impairment in SCFA production (237). Though the authors observed a reduction in butyrate-producing *Roseburia hominis* and *F. prausnitzii*, as well as a reduction in butyrate levels, they could not draw a direct correlation between these findings. This provides evidence that the microbiota may not only be divergent regarding the abundance of community members but also in their metabolic activity.

Furthermore, the definition of dysbiosis should not be a mere one-sided microbial consideration but should take the host into account as well. Palm et al. showed that IBD patients display an *altered immune recognition of a dysbiotic microbiota* correlating with an increased and divergent IgA coating (75). By using an animal model of chemically induced colitis, they also transferred the disease susceptibility and thereby proved the true causal role of this IgA-coated ecosystem. Consequently, dysbiosis may also be defined as an “alteration of symbiosis” with pathophysiologic consequence (257).

## What Causes Dysbiosis?

**Figure 1:**
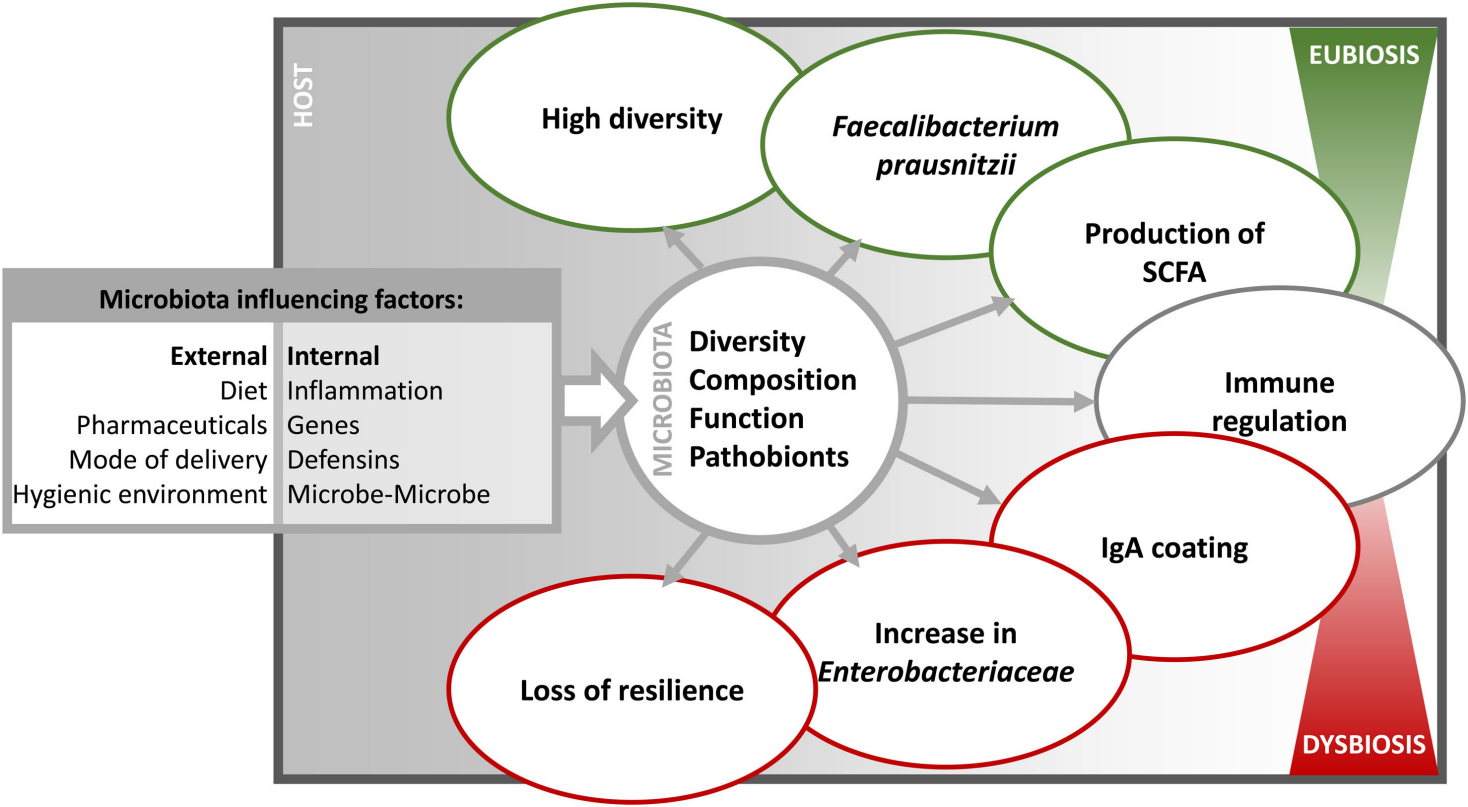
**The different influencing factors and characteristics of dysbiosis**. The intestinal microbiota is influenced in diversity, composition, function, and prevalence of pathobionts by internal and external factors. Consequently, the microbiota varies in immune relevant characteristics and thereby shifting from a eubiotic to a dysbiotic state.

The sum of environmental triggers, so-called exposome, including diet, early nutrition (breast or formula feeding), mode of delivery, hygienic milieu (contact to disinfectants or animals), stress, drugs, lifestyle, and geography (e.g., air pollution and location) may contribute to shape the intestinal ecosystem (Figure 1) (185, 208, 258–266). Diet has an enormous impact on the intestinal ecology. For instance, diet high in fat shifts the microbiota toward a more dysbiotic pattern associated with increased risk of intestinal inflammation. David et al. effectively showed the plasticity and the stability of the intestinal ecosystem upon short-term perturbations by feeding mice an exclusively animal- or plant-based diet (267). Animal-based diet promotes bile-tolerant microorganisms, such as *Alistipes* or *Bilophila* (194, 267). For instance, the increase in *Bilophila* is of interest considering that *Bilophila wadsworthia* was previously shown to exert pathobiont behavior in IL-10^−/−^ (268). In another study, a western diet (rich in fat and sugar) induced dysbiosis and favored the increase in AIEC LF82 upon colonization in genetic susceptible CEABAC10 mice (269). Several other studies showed that dramatic shifts in the intestinal ecosystem are not induced by short-term changes in dietary habits or application of single nutrient but require long-term pressure on the ecosystem (189, 270).

Beside exposomal factors, also *intrinsic factors*, i.e., host genetic, shape the intestinal microbiota. Several genes associated with altered immune function, microbial recognition, or antimicrobial defense were shown to influence the intestinal ecosystem. In a meta-analysis, Knights et al. highlighted the significant association between the NOD2 risk allele and increased abundance of *Enterobacteriaceae* in IBD patients (271). This observation was backed up by cohousing experiments of NOD2-deficient mice which develop a genotype-induced dysbiosis. By transferring this dysbiotic microbiota to WT cage-mates, they became more susceptible to DSS colitis (130). In another study, Rausch et al. showed an association between the intestinal microbiota and the FUT2 (secretor) gene – a physiological trait that regulates gastrointestinal mucosal expression of blood group A and B antigens. Approximately 20% of humans lack the FUT2 gene, which was shown to be a risk factor for CD development (272). Within the group of CD patients, the microbiota from FUT2 carriers differed from non-secretors in composition, diversity, and functionality (245, 273).

In intestinal pathologies, such as IBD, dysbiosis is associated to inflammation. It has been proposed that the shift in bacterial composition is due to the inability of some bacterial taxa to adapt to the inflammatory milieu characterized by increased secretion of AMPs (274). The potential of AMPs to shape the intestinal ecology and their importance in IBD has been shown by others before (275). The inflammatory process may also induce stress or upregulation of virulence-associated genes in the microorganism, as shown in monoassociation studies with the gut commensal strains *E. faecalis* OG1RF (276) or *E. coli* NC101 (277, 278). For instance, metalloprotease gelatinase E (GelE) secreted by *E. faecalis* contributes to the development of chronic intestinal inflammation in mice that are susceptible to intestinal inflammation (IL-10^−/−^ and TNF^deltaARE^ mice) by impairing epithelial barrier integrity (279). *E. faecalis* isogenic mutants lacking GelE, enterococcal polysaccharide antigen, or lipoproteins (Lgt) show significantly decreased inflammation in the distal colon of monoassociated IL-10^−/−^ mice and impaired TLR2-mediated activation of DCs (276, 279). *E. coli* NC101 cells isolated from inflamed IL-10^−/−^ mice displayed upregulated bacterial-stress response genes (e.g., heat shock proteins) compared to isolates from WT mice (277, 278). However, inflammation may also promote growth advantages and virulence of pathogens. For *S. typhimurium*, it was shown that the host produces tetrathionate under inflammatory circumstances, which consequently promotes the ethanolamine utilization of *S. typhimurium* and thereby its colonization fitness (280, 281). Also nitrate produced by the host during inflammatory processes is assumed to promote growth of *Enterobacteriaceae*. Consequently, the often discussed increase of *Enterobacteriaceae* in IBD may also regard as secondary to inflammatory processes rather than causative (282).

Apart from host-derived factors, also microbe-derived mechanisms exert impact on other commensals. The transfer of single strain or bacterial consortia to GF mice enables to study mechanisms of *microbe–microbe interaction*. Commensals are capable of inhibiting growth of pathogens by, e.g., competition for nutrients, quorum sensing-mediated colonization repression, as well as expression of virulence factors (220, 283–285). Metabolic interaction (food chain) of commensals and pathobionts are also shaping the intestinal ecosystem, as some organisms depend on the conversion of dietary components or induction of host-derived nutrients by other members of the microbiota (217, 286–288). Also quorum sensing-mediated repression of colonization is regarded as an important mechanism of shaping the intestinal ecosystem, and recent findings from Thompson et al. demonstrate the role of autoinducer-2 in reshaping antibiotic-induced dysbiosis in the gut (284, 289).

## The Role of the Microbiota in Animal Models of Intestinal Inflammation

Most dysbiosis-influencing factors cannot be studied under highly controlled and standardized conditions in humans. To discover causative dysbiotic shifts in the microbiota prior to disease onset, large prospective cohort screenings would be necessary, which are hardly feasible due to low incidence rates. Therefore, animal models of intestinal inflammation are gaining more and more importance, as confounders like diet, genetic background, and hygienic environment can be continuously controlled. *Genetically driven animal models* developing inflammation without any chemical inductor (e.g., DSS or TNBS) are especially appropriate to study the interaction of the intestinal microbiota and pathology, as the chemical compound *per se* would influence the ecosystem. Ever since the availability of *GF and gnotobiotic animal models*, great progress has been made to portray microbe–host interaction. Hereby, many IBD mouse models were shown to be free of disease in GF conditions and thereby clearly prove the causality of microbial triggers in IBD development.

The IL-10^−/−^ mouse – incapable of producing the anti-inflammatory cytokine IL-10 – is the best-studied model of colitis. In GF housing, IL-10^−/−^ mice do not develop inflammation, and interestingly the time point of microbial colonization seems to be of importance, as IL-10^−/−^ mice colonized at adult age developed more severe inflammation (182). A more recent model of UC is the Rag2^−/−^ × Tbx21^−/−^ (TRUC) mouse model. Those mice develop UC-like colitis due to the absence of an adaptive immune system. TRUC mice were free of colitis in GF conditions as well (290). Another model protected from inflammation under GF conditions is the TCR alpha-knockout model. These mice are characterized by a defective immunity as well, as αß T cells are absent (291). Also severe combined immunodeficiency (SCID) mice, having an impaired B and T cell functions, are free of colitis in GF-housing conditions (292).

However, all the above-mentioned models display a colitis phenotype. To study mechanisms relevant in CD, which is characterized by ileal involvement, just few animal models are available. The SAMP1/YitFc mouse model is one of the rare models displaying a CD-like inflammation, with still uncertain mechanism of pathology induction (293, 294). Nevertheless, a recent study suggests that inflammation may be mediated by loss of CCL21 signaling and DC migration from the ileal lamina propria to mesenteric lymph nodes (293–297). Another model of CD-like ileitis is the TNF^deltaARE^ mouse model. These mice have increased TNF levels due to a deletion of the adenosine–uracil-rich element in the TNF transcript (298). The pathology in TNF^deltaARE^ mice with their transmural ileitis and the Th-1-driven immune response give a very good resemblance to the inflammation in CD patients. In line with this, TNF antibodies, which are a treatment option in CD patients, were shown to hamper inflammation in TNF^deltaARE^ mice (299). Just recently, the necessity of a microbial trigger for inflammation development was demonstrated, as TNF^deltaARE^ mice are free of inflammation in GF housing (181). In contrast to SAMP1/YitFc mice, the inflammation in TNF^deltaARE^ mice is not characterized by the formation of “cobblestone” structures – a thickening of the gut wall with protruding lesions found also in some patients with progressive IBD in humans (300, 301). These divergent three-dimensional structural inflammatory phenotypes again highlight the necessity and basic requirement of different mouse models to study the mechanisms of inflammation development and to be able to characterize the complexity of inflammation in IBD.

Mice with a conditional deletion of caspase 8 (a protease involved in apoptosis regulation) in the IECs (Casp8^deltaIEC^ mice) spontaneously develop chronic inflammation in the terminal ileum due to increased necroptosis (302). Notably, antibiotic-treated Casp8^deltaIEC^ mice were rescued from ileitis, indicating an important role for the intestinal microbiota in the development of the disease (303).

Finally, the development of animal models that mirror human genetic risk factors for IBD or other pathologies may be an important step toward unraveling mechanisms of pathology. The disease-free status of animal models in GF housing is exploited as valuable tool to investigate the development of dysbiosis, its influencing factors, and also mechanisms of microbe–host interaction in gnotobiotic setups.

## Dysbiosis and Microbe–Host Interaction in Gnotobiotic Animal Models

Specific mechanisms of disease induction can be studied through the colonization of GF mice with single bacterial strain. Therefore, many colonization studies have been performed for models of intestinal inflammation as well as for other pathologies, including obesity, diabetes, and multiple sclerosis, as recently summarized (304). However, by colonizing immunocompromised GF animals with selected single strain, several obstacles are met. Not all microbes colonize equally, and the induction of inflammation depicts bacteria as well as model-specific traits.

In the IL-10^−/−^ model, several monoassociations with candidate pathobionts failed to induce inflammation, such as *Helicobacter hepaticus* and *E. coli*, while other bacteria were effective in developing pathology, such as *E. faecalis* or *B. wadsworthia* (279, 305, 306). These findings suggest that an association with only one bacterial strain may not be sufficient to induce the necessary immune maturation required for the establishment of the inflammatory status. Atarashi et al. could show that one strain of *Clostridium* was not sufficient to induce regulatory immune functions in former GF animals, as this relies on concerted actions of different strains (307, 308). It is tempting to speculate that not all members of the microbiota are equally sufficient to induce pathology but rather requires the interaction with other commensals. An example of this hypothesis encompasses monoassociation of IL-10^−/−^ mice with *H. hepaticus* or *Lactobacillus reuteri*, which as single strain did not induce inflammation, while the combination of the two bacteria induced colitis (306). Therefore, current studies tend to use complex microbial settings or defined microbial consortia to ensure sufficient immune maturation in GF animals. Garrett et al. showed that colitis in TRUC mice correlates with increased abundance in *Klebsiella pneumonia* and *Proteus mirabilis*. However, the combined colonization of TRUC or Rag2^−/−^ recipient mice with both these two bacterial strains did not induce inflammation. Just a combined colonization of *K. pneumonia*, *P. mirabilis*, and a complex microbiota induced inflammation in recipient Rag2^−/−^ mice (290). However, it has to be mentioned that WT cage-mates were also inflamed, suggesting a rather pathogenic than dysbiotic trait of the microbiota. Interestingly, Powell et al. identified *Helicobacter typhlonius* as a key driver of pathogenesis in TRUC mice (309). The transfer of a complex microbiota from inflamed TRUC mice induced colitis in gnotobiotic TRUC mice as well. However, when the microbiota from antibiotic-treated mice in remission was transferred, the recipients developed only attenuated inflammation (310). In the SCID mouse model, Stepankova et al. showed inflammation development by segmented filamentous bacteria (SFB) only in combination with complex specific pathogen-free (SPF) microbiota (292). SFB are known to be potent inducers of the host’s immune response as summarized elsewhere (292, 311). This also points out the fact that monocolonizations may pose elegant ways to prove causality, whereas complex mechanisms of disease initiation may need more complex interactions. TNF^deltaARE^ mice colonized with the pathobiont *E. coli* LF82 did not develop ileitis, whereas the disease was induced upon colonization with microbiota from inflamed donors (181). Those mice showed in SPF housing high variance in ileitis development due to spontaneous shifts in the intestinal microbiota, i.e., inflamed mice displayed microbial patterns distinct from non-inflamed mice. By transferring the microbiota from inflamed TNF^deltaARE^ to GF mice, the TNF^deltaARE^ recipients develop inflammation. The WT cage-mates did not develop inflammation, showing that the microbiota was truly dysbiotic and not pathogenic, as susceptibility of the host is needed for inflammation development. By transferring the microbiota from non-inflamed TNF^deltaARE^ mice, no inflammation was observed in TNF^deltaARE^ or WT recipients. This clearly pinpoints that the concerted action of a complex microbiota with dysbiotic patterns and the genetic susceptibility are precondition for the CD-like pathology in this model.

The studies mentioned in this review clearly emphasize the role of dysbiosis in intestinal pathology, as well as its multifactorial etiology. Until now, no single microbial trait could be identified as a clear indicator of a dysbiotic state itself. It was rather shown that dysbiosis is characterized by multifaceted variations in community networks and displays specificity for the respective host and inflammatory condition. Several human studies reported dysbiosis before onset of disease (234, 312). In animal models of intestinal inflammation, the causal relationship of a dysbiotic ecosystem in inducing inflammation was effectively shown by transferring the microbiota directly or by cohousing animals and thereby inducing pathology in recipients (130, 181, 290, 310). In these animal models which have different mechanisms of pathology induction, a single microorganism is not capable to induce tissue inflammation but needs the concerted action of a complex microbiota. These observations are further supported by the fact that no single pathobiont has been found that is consistently present in different IBD cohorts. More and more studies show that dysbiosis is characterized by changes in the microbial composition and microbial function rather than mere reduction in diversity or expansion of pathobionts. This may also explain why by now, no single probiotic treatment was found able to restore dysbiosis by administration of just one single probiotic strain, suggesting that one single species may not be powerful enough revert complex dysbiotic shifts.

## Rebiosis as Concept for Clinical Therapy

Even though a high number of studies have been performed on the benefits of probiotics in IBD, new meta-analyses show no overwhelming or placebo-superior effect (313, 314). Strain-specific mechanisms were investigated showing the potential of probiotics to enhance intestinal barrier integrity, to counteract proinflammatory cytokines, or to induce an anti-inflammatory immune response (315–317). Newer studies go from single probiotic strain or mixtures with low defined numbers to more complex setups. In *C. difficile*-induced infection, fecal microbiota transplantation (FMT) seems to be very promising and superior to antibiotic treatment regimens (318–322). Hereby, the complex fecal microbiota of a healthy donor (often a relative or spouse of the patient) is processed, standardized, and subsequently transferred to the patient by either nasogastric route or colonoscopy. Although the bacterial taxa associated with a successful FMT-donor microbiota have not been discovered yet, the subsequent increase in the intestinal microbial diversity of the recipient is in most cases associated with recovery (323). However, due to the novelty of this treatment approach, appropriate cohort numbers and well-controlled trials are to be provided in the future. The generation of biobanks for frozen donor microbiota (e.g., OpenBiome; Microbiome Health Research Institute Inc.) is of utmost importance. A future prospect is FMT capsules for therapeutic treatment of infectious diseases, such as *C. difficile* infection, which is currently under development (324). Despite the fact that FMT was highly effective in *C. difficile* infection, its therapeutic implementation in IBD is unclear. A recent study by Moayyedi et al. showed a superior placebo effect of FMT in UC patients, though this effect is donor and time dependent (325). An overall analysis of uncontrolled studies with small patient cohorts shows limited success for FMT as standard therapy in IBD, and the rationale of introducing new antigen pools in a milieu that has been overreacting to microbial stimuli is questionable (326, 327).

The causality of dysbiosis in IBD development has been reported, but clear compositional patterns and functionality are still to be unraveled. These aspects are of importance for basic understanding of which consortia may be effective for therapeutic potential. Finally, the intestinal bacterial community is under the influence of environmental-, host-, and microbial-derived factors, and therefore, microbial composition is not the only trait to be considered in order to choose the suitable donor, but also the recipient genetic background and the exposome should be taken into consideration.

## Conclusion and Outlook

Despite a growing body of evidence suggesting a causative role of the intestinal microbiota in CD pathogenesis, the impact of bacteria on disease progression, the development of the various disease phenotypes, the risk to develop therapy refractoriness, or the risk to relapse is completely unclear. An increasing number of sequencing studies in human cohorts show associations of certain bacterial groups and CD development. However, due to the nature of the study or high interindividual variation, they often lack the possibility to depict mechanisms causal for disease initiation. Therefore, animal models of intestinal inflammation are important to show functionality and to depict characteristics of dysbiosis. The possibility to unravel certain mechanisms of interaction in animal models of inflammation is generating important insights. The role of antigenic surface compounds or microbial-derived metabolites is of great interest to elucidate mechanisms of microbiota-induced shifts in intestinal homeostasis. Even though new mouse models were recently generated which display more hypothesis-driven mechanisms of intestinal inflammation, it is evident the paucity of model organisms that accurately reproduce all aspects of multifactorial disease, such as CD.

Questions remain about the reliability of mouse models as tool to draw interpretations on human disease. In some cases, mouse research has led to major advantages in the ability to treat serious conditions. For instance, work on the acute promyelocytic leukemia mouse model resulted in successful treatment for this type of cancer in human patients (328). Knocking out the leptin gene in mice manifested the role this hormone has in regulating appetite and, by extension, preventing obesity (329). In contrast, mice are not always reliable as models for human disease as shown in the case of specific IL-17-deficient mouse strains which generated conflicting results in preclinical models of IBD (330).

Similar questions arise from the use of humanized mice in order to unravel human molecular mechanisms or functions. The humanization process consists in repopulation of mice with human hematopoietic cells or in colonization of the gut of GF mice with human microbiota. The potential of these tools for the study of human immune function and causality of the complex host–microbiota interactions *in vivo* is apparent. Nevertheless, it should be taken into consideration whether the mouse system is able to support development of human immune cells and whether antigens from human origin enable murine immune system maturation. Additionally, it has been shown before that bacterial strains sharing similar genomic content exert differential ability to stimulate the host response (331, 332), suggesting that molecular and biochemical differences in cell envelope architecture may account for the variation in cytokine profiles. This evidence leads to the hypothesis that some bacterial strains may harbor host specificity, and therefore, isolates specific for the human setup might not be able to induce maturation of the mouse immune system, as previously suggested (333).

Finally, it should be noted that even though GWAS have linked genetic variations to several human conditions, they do not provide information on their functions. Animal models are clearly a fundamental tool to identify gene function, and how mutations in gene associated to the disease alter these functions.

## Conflict of Interest Statement

The authors declare that the research was conducted in the absence of any commercial or financial relationships that could be construed as a potential conflict of interest.
